# Introduction of New Dengue Virus Lineages of Multiple Serotypes after COVID-19 Pandemic, Nicaragua, 2022

**DOI:** 10.3201/eid3006.231553

**Published:** 2024-06

**Authors:** Cristhiam Cerpas, Gerald Vásquez, Hanny Moreira, Jose G. Juarez, Josefina Coloma, Eva Harris, Shannon N. Bennett, Ángel Balmaseda

**Affiliations:** Sustainable Sciences Institute, Managua, Nicaragua (C. Cerpas, G. Vásquez, H. Moreira, J.G. Juarez, Á. Balmaseda);; Laboratorio Nacional de Virologia, Centro Nacional de Diagnóstico y Referencia Ministerio de Salud, Managua (C. Cerpas, Á. Balmaseda);; Division of Infectious Diseases and Vaccinology, School of Public Health, University of California Berkeley, Berkeley, California, USA (J. Coloma, E. Harris);; California Academy of Sciences, San Francisco, California, USA (S.N. Bennett)

**Keywords:** dengue, neglected disease, dengue virus, genome, serotypes, viruses, parasites, vector-borne infections, Nicaragua

## Abstract

Major dengue epidemics throughout Nicaragua’s history have been dominated by 1 of 4 dengue virus serotypes (DENV-1–4). To examine serotypes during the dengue epidemic in Nicaragua in 2022, we performed real-time genomic surveillance in-country and documented cocirculation of all 4 serotypes. We observed a shift toward co-dominance of DENV-1 and DENV-4 over previously dominant DENV-2. By analyzing 135 new full-length DENV sequences, we found that introductions underlay the resurgence: DENV-1 clustered with viruses from Ecuador in 2014 rather than those previously seen in Nicaragua; DENV-3, which last circulated locally in 2014, grouped instead with Southeast Asia strains expanding into Florida and Cuba in 2022; and new DENV-4 strains clustered within a South America lineage spreading to Florida in 2022. In contrast, DENV-2 persisted from the formerly dominant Nicaragua clade. We posit that the resurgence emerged from travel after the COVID-19 pandemic and that the resultant intensifying hyperendemicity could affect future dengue immunity and severity.

Arthropodborne viruses are distinguished by alternating transmission between arthropod vectors and vertebrates. Some of the most clinically relevant arboviruses (e.g., dengue, Zika, and yellow fever) belong to the genus *Flavivirus*. Dengue virus (DENV) alone threatens more than half the world’s population (*1*). DENV, transmitted by female *Aedes* mosquitoes, consists of 4 serotypes, DENV-1–4. Each serotype comprises 4–6 major lineages (genotypes), composed of various sublineages or clades (*2*). Clinical manifestations of infection may vary by DENV serotype, genotype, and clade (*3*–*6*); infection outcome (asymptomatic to potentially fatal disease) is also modulated by prior immunity. Knowledge of the complexities of DENV serotype, genotype, and specific lineage circulation is critical for improving public health responses to outbreaks.

Dengue epidemics cycle every 2–5 years in tropical and subtropical regions and are strongly influenced by season (*7*). In many countries, increasing human mobility, societal changes affecting human–mosquito contact, and challenges with vector control have all contributed to increased dengue epidemic activity and a change from nonendemic or hypoendemic patterns (noncontinuous transmission of a single serotype) to hyperendemic transmission patterns (multiple cocirculating serotypes) (*8*), resulting in increased interannual duration of transmission activity with fewer intervening years of little to no transmission.

DENV was first detected in Nicaragua in 1985 (*9*); since then, the 4 serotypes have periodically circulated in the country, albeit differing in relative and overall prevalence (*5*,*9*–*12*). Dengue has become a serious public health concern in Nicaragua because of its incidence and potential for severe and possibly fatal disease (*10*,*13*,*14*). We investigated the molecular and epidemiologic characteristics of the 2022 epidemic after air travel resumed and neighboring countries fully reopened their borders after the COVID-19 pandemic. We observed substantial cocirculation of all 4 DENV serotypes, including DENV-4 after a 30-year absence of epidemic transmission. We generated virus genome sequences in Nicaragua using a portable genomic approach, enabling a detailed description of DENV evolutionary histories and demonstrating the introduction of new clades of 3 serotypes (DENV-1, DENV-3, and DENV-4) and persistence of DENV-2.

## Materials and Methods

### Ethics Statement

Protocols for the collection and testing of samples were reviewed and approved by the institutional review board of the University of California, Berkeley (PDCS: 2010-09-2245; PDHS: 2010-06-1649; A2CARES: 2021-03-14191). They were also approved by the institutional review board of the Nicaraguan Ministry of Health (PDCS: CIRE 09/03/07/-008.Ver25; PDHS: CIRE 01/10/06-13. Ver. 18; A2CARES: CIRE 02/08/21-114 Ver. 4).

### Study Population and Sample Collection

Samples were collected during June–December 2022 as part of 3 ongoing studies, the Nicaraguan Pediatric Dengue Cohort Study (PDCS), Pediatric Hospital-based Study (PDHS), and Asian-American Center for Arbovirus Research and Enhanced Surveillance (A2CARES) cohort study (part of the National Institutes of Health Centers for Research in Emerging Infectious Diseases Network), and through the National Dengue Surveillance Program of the Nicaraguan Ministry of Health (Appendix Table). Since 2004, the PDCS has been conducted within the Sócrates Flores Vivas Health Center catchment area in District 2 of Managua, with participants 2–17 years of age (*3*,*13*,*15*,*16*). The PDHS collects samples from patients with suspected dengue 6 months–14 years of age who receive medical attention in the Infectious Diseases Ward of the National Pediatric Reference Hospital (Hospital Infantil Manuel de Jesús Rivera) in Managua and who have received parent/guardian consent for the study (*17*). The A2CARES cohort study follows ≈2,000 persons 2–80 years of age in ≈1,000 households in District 3 of Managua.

We selected 3,171 suspected dengue cases for potential sequencing analysis, of which 1,353 were confirmed as DENV-positive with known serotype. To ensure a representative national distribution in the selection of samples for sequencing, we included 10 ±2% of the DENV-confirmed serotyped samples from each department that had adequate coverage for genomic analysis (n = 135), which included samples from the studies in Managua. We sequenced 49 DENV-1, 6 DENV-2, 38 DENV-3 and 42 DENV-4 samples that were positive by real-time reverse transcription PCR (rRT-PCR). To assess the relationships of the samples collected in 2022 to previously circulating strains from Nicaragua, we included 68 DENV-1, 62 DENV-2, and 51 DENV-3 previously published sequences (*5*,*10*,*18*).

### RNA Extraction and RT-PCR

Serum or plasma collected during the acute phase (days 1–5 since symptom onset) from patients with suspected dengue was processed at the National Virology Laboratory (Centro Nacional de Diagnóstico y Referencia, Ministerio de Salud, Managua, Nicaragua). We extracted viral RNA by using the QIAmp viral RNA mini kit (QIAGEN, https://www.qiagen.com) according to manufacturer instructions and used it to perform a single-reaction multiplex rRT-PCR for detection of Zika virus, chikungunya virus, and DENV (*19*). We further analyzed DENV-positive samples to identify the infecting serotype by using a multiplex DENV rRT-PCR (*20*,*21*). We prioritized cycle threshold values <28 for genomic sequencing to ensure the presence of ample genetic material, which ultimately enhanced sequence quality, minimized contamination risk, and improved detection sensitivity. In >90% of cases, sequences included in genomic analysis had >60% genome coverage.

### Library Preparation and Next-Generation Sequencing

We reverse transcribed sample RNA (8 μL) with 2 μL LunaScript RT SuperMix (New England Biolabs, Inc., https://www.neb.com) as follows: 25°C for 2 minutes, 55°C for 10 minutes, 95°C for 1 minute, and a 4°C hold. The PCR reaction mixture contained 13.75 μL of nuclease-free water, 1.5 μL of pool A and pool B primers, 5 μL of 5X Q5 reaction buffer, 2 μL of 2.5 mM dNTPs (Invitrogen, https://www.thermofisher.com), 0.25 μL of Q5 DNA polymerase, and 2.5 μL of sample cDNA (*22*,*23*). PCR amplification consisted of 98°C for 30 seconds, followed by 45 cycles of 98°C for 15 seconds and 65°C for 5 minutes, followed by a 12°C hold. We selected samples with a 900-bp band in the pool A and pool B mix for sequencing. Using 5 μL pooled PCR products (from pools A and B), we prepared the cDNA MinION library by using a native barcoding kit (NBD196; NgelabKampus, https://ngelabkampus.com) with a ligation sequencing kit (LSK-109; Interprise, https://interpriseusa.com) at 30°C for 2 minutes and 80°C for 2 minutes according to manufacturer instructions. We cleaned up PCR products by using AmpureXP purification beads (Beckman Coulter Diagnostics, https://www.beckmancoulter.com) and loaded the DNA library onto a primed MinION flow cell R9.4 (FLO-MIN 106; Oxford Nanopore Technologies, https//nanoporetech.com).

### Consensus Genomes

We extracted raw sequence reads by using filters for quality and trimmed adaptors by using Porechop version 0.2.3_seqan 2.1.1 (https://github.com/rrwick/Porechop) and assessed resulting sequence quality by using Nanoplot (*24*). We mapped raw reads by using Minimap2 v2.17-R941 (https://github.com/lh3/minimap2) against reference genomes from GenBank for DENV-1 (accession no. NC_001474), DENV-2 (accession no. NC_001474.2), DENV-3 (accession no. NC_001475.2), and DENV-4 (accession no. NC_002640.1) by using the pipeline at https://github.com/gvaleman/CONSENSO_D. We generated consensus sequences by using SAMtools version 1.7 (https://www.htslib.org/doc/1.7/samtools.html) with the default minimum depth coverage of 1 and confirmed the serotype by using Genome Detective Arbovirus Typing software version 4.1 (https://www.genomedetective.com) and BLAST (https://blast.ncbi.nlm.nih.gov). Mean depth coverage by serotype per SAMtools-depth tool was 221 for DENV-1, 68 for DENV-2, 516 for DENV-3, and 393 for DENV-4.

### Phylogenetic Analyses

To increase the resolution of the genetic relatedness of the 2022 viruses in terms of genotype, country, and time of sample origin, we constructed alignments with initial broad coverage by using the National Center for Biotechnology Information Virus database (https://www.ncbi.nlm.nih.gov/labs/virus) by first subjecting sequences to BLAST and selecting the top 100 hits, followed by performing a directed search across regions and timescales represented in the database. Thus, we cast a broad net of potential related sequences. To reduce redundancy while maintaining representation (genotype, country, year) for final analysis, we aligned the large sample sets and reviewed them as initial RAxML (*25*) trees (Appendix Figures 1–4) before downsampling. We conducted sequence alignments with MAFFT version 7.4 (*26*) and manually reviewed them by using AliView version 1.28 (https://ormbunkar.se). We constructed maximum-likelihood phylogenetic trees for each serotype by using RAxML-HPC Blackbox version 8.2.12 and using a general time-reversible  substitution model with bootstrap replicates determined automatically. We visualized the maximum-likelihood trees by using FigTree version 1.4.4 (http://tree.bio.ed.ac.uk) (data not shown). We performed Bayesian phylogenetic analysis by using BEAST version 1.10.4 (https://beast.community) and a Hasegawa-Kishino-Yano substitution model, a less parameterized model (2 substitution rates, for transitions and transversions) often used in place of the general time-reversible model above (6 substitution rates, for every base combination) to reduce computational burden. We also used a strict molecular clock and a Bayesian constant size coalescent tree. We ran the Markov chain Monte Carlo analysis for 100 million steps, taking samples every 5,000 steps, discarding the first 10% as burn-in. We assessed convergence with Tracer version 1.7.1 (http://tree.bio.ed.ac.uk) by using an effective sample size threshold >200. We assessed parameter estimate uncertainty by using 95% high probability density (HPD) intervals. Last, we generated maximum clade credibility (MCC) trees by using TreeAnnotator version 1.10 (https://beast.community) and visualized them with Figtree version 1.4.4.

**Figure 1:**
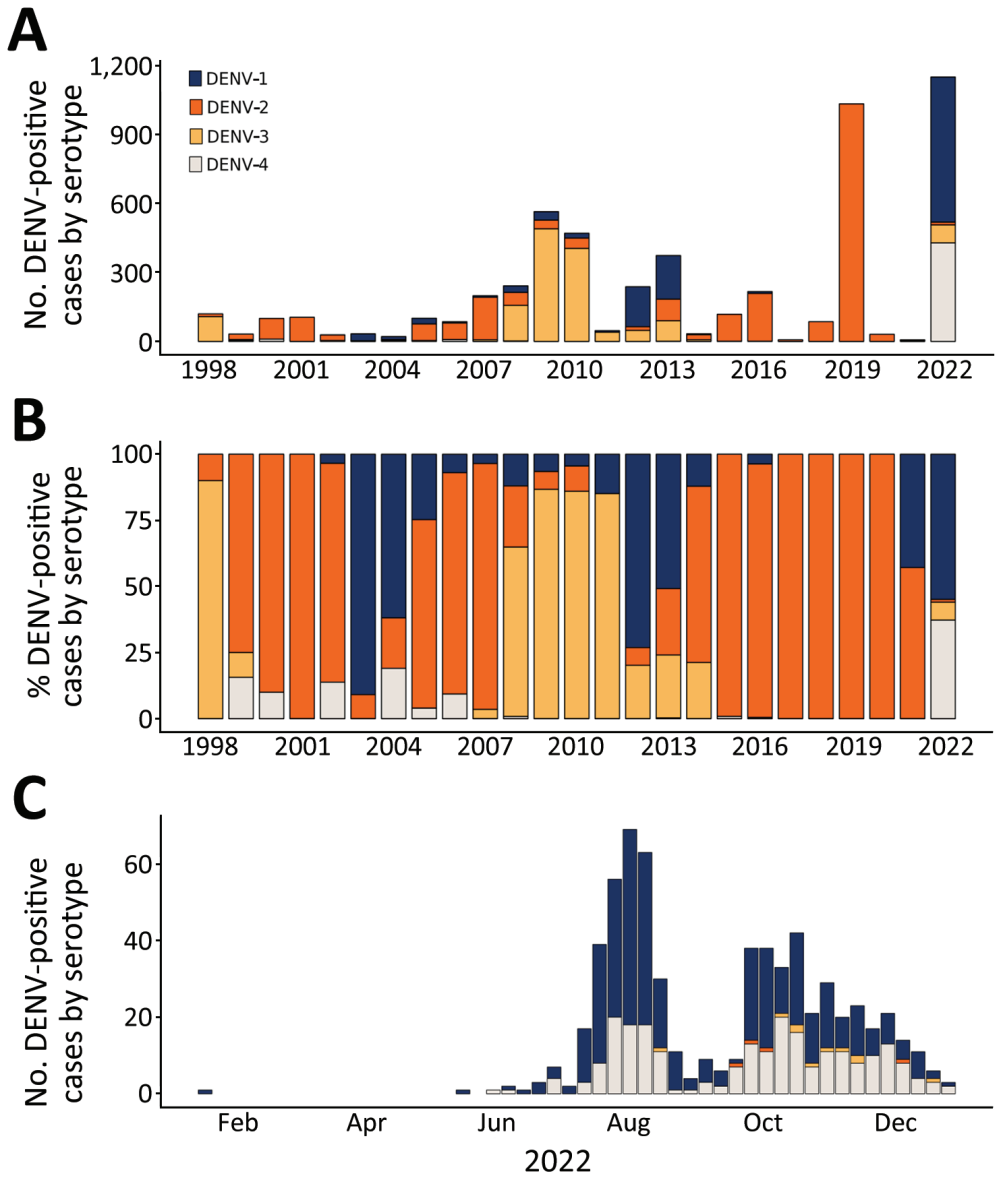
DENV serotypes 1–4 circulation in Nicaragua during 1998–2022. Data on dengue cases with serotype information available were obtained from the Nicaraguan Dengue Surveillance Program; many more cases were confirmed via serology that are not included here because the serotype is unknown. A) Total dengue cases by serotype and year. B) Percentage of circulating dengue cases by serotype and year. C) Case count of each serotype identified by real-time reverse transcription PCR over time during January–December 2022. DENV, dengue virus.

**Figure 4:**
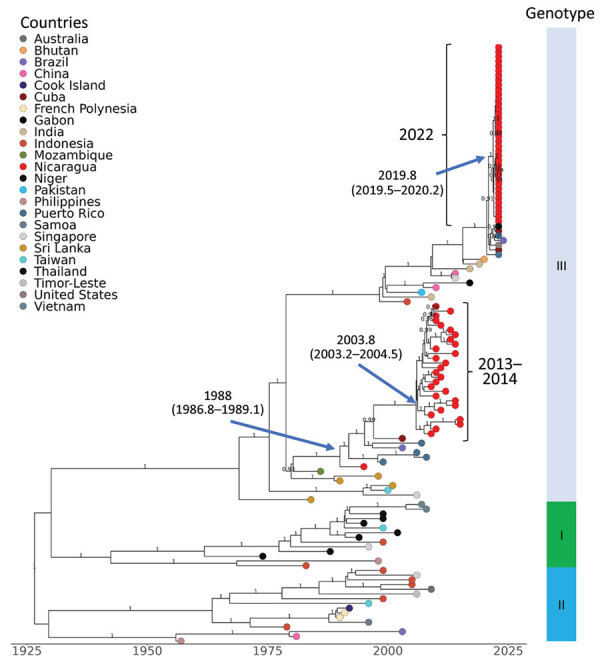
Dengue virus (DENV) serotype 3 maximum clade credibility tree, generated from 131 DENV-3 nucleotide sequences, showing emergence over time. Sequences are labeled by country of sample origin, indicated by colored circles, and include Nicaraguan sequences (red) and publicly available sequences, as indicated. Nicaragua clades are indicated with square brackets labeled by year(s) of detection. The estimated time to most recent common ancestor (95% high probability density) is indicated at the nodes leading to Nicaraguan clades. Genotypes are identified in the vertical bar to the right. Posterior node probabilities are indicated at major nodes.

## Results

### Dengue Epidemiology after the COVID-19 Pandemic

Dengue epidemics in Nicaragua typically occur every 2–3 years (Figure 1, panel A); a given serotype surges to epidemic proportions roughly every 10 years: a shift from DENV-3 in 1998 (*14*) to DENV-2 in 1999 (*3*,*13*,*27*) to DENV-1 in 2003 (*3*) and again to DENV-2 in 2007 (*5*), then another surge of DENV-3 in 2009–11 (*28*), DENV-1 in 2012–13 (*10*), DENV-2 in 2015 and 2019 (*11*,*18*), and the cocirculation of all 4 serotypes in 2022 (Figure 1, panels A, B). However, during 2015–2020, only DENV-2 was detected in patients, whereas large epidemics resulted from newly introduced arboviruses such as chikungunya and Zika, followed by SARS-CoV-2 (*29*–*31*). Although Nicaragua did not implement official lockdown measures in response to the COVID-19 pandemic, international and specifically airline travel was severely curtailed for at least 1 year, starting in May 2020 (*32*,*33*). Together, the prolonged dominance of DENV-2 and the limited movement of people during the pandemic created a window of opportunity for hyperendemic DENV transmission in 2022 because of the susceptibility of the population to other serotypes. Furthermore, the circulation of Zika in 2016 might have primed immunity to increase DENV-4 cases (*12*). The result was the cocirculation of all 4 serotypes in 2022 (Figure 1, panel C), associated with an ≈100-fold increase in reported dengue cases from the previous year.

### DENV Genomic Surveillance during the 2022 Epidemic

Serum/plasma samples targeted for sequencing were collected through the PDCS (n = 57), PDHS (n = 13), A2CARES cohort study (n = 4), and the Nicaragua epidemiologic surveillance system (n = 61) (Appendix Table) (*16*,*17*). A total of 135 sequences were generated by using newly established in-country sequencing capability based on the MinION platform and in-house bioinformatics and phylogenetics pipelines, markedly accelerating the time from clinical sample collection to pathogen sequencing from months to days.

### DENV-1

**Figure 2:**
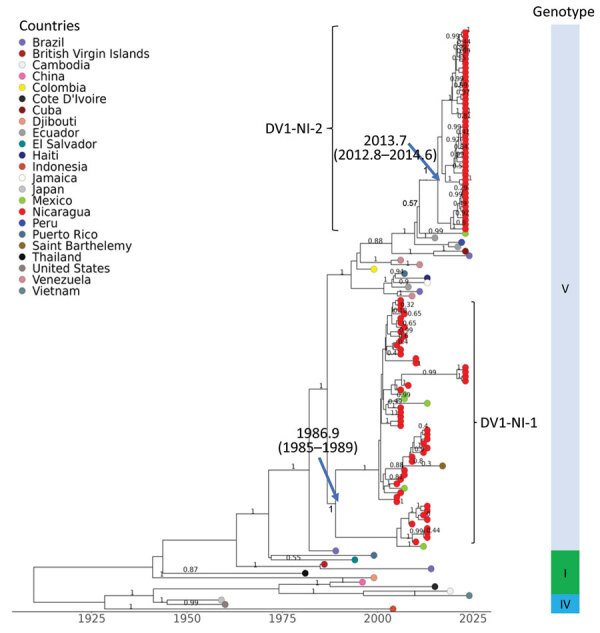
Dengue virus (DENV) serotype 1 maximum clade credibility tree, generated from 130 DENV-1 nucleotide sequences, showing emergence over time. Sequences are labeled by country of sample origin, indicated by colored circles, and include Nicaragua sequences (red) and publicly available sequences. DENV-1 genotypes are identified in the vertical bar to the right. Two Nicaragua clades, DV1-NI-1 and DV1-NI-2, are indicated with square brackets. The time to most recent common ancestor (95% high probability density range) is indicated at the nodes leading to Nicaragua clades. Posterior node probabilities are indicated at major nodes.

We used 49 DENV-1 sequences from our study along with 81 publicly available sequences to build phylogenetic trees by using maximum-likelihood and Bayesian Markov chain Monte Carlo methods to generate an MCC tree (Figure 2; Appendix Figure 1, showing the initial ML tree with 408 taxa). DENV-1 genomes recovered in 2022 all belong to genotype V (American/East African and Asian genotype), consistent with previous studies of DENV-1 in Nicaragua (*10*). However, the viruses from 2022 clustered into 2 distinct clades, DV1-NI-1 and DV1-NI-2. Most sequences clustered into DV1-NI-2, for which the closest relatives were Ecuador sequences from 2014, suggesting that DV1-NI-2 is derived from an introduction from South America into Nicaragua. The other clade, DV1-NI-1, is represented by only 4 sequences and is associated with strains previously circulating in Nicaragua in 2013 and 2016. The date estimate of the time to most recent common ancestor (tMRCA) for DV1-NI-2 was 2013.7 (95% HPD 2012.8–2014.6) and for DV1-NI-1 was 1986.9 (95% HPD 1985–1989) (Figure 2). The genetic distance between DV1-NI-2 (2022) and DV1-NI-1 (2013 and 2016) is substantial, based on branch lengths relative to the rest of the phylogeny; mean rate is 0.04 substitutions per site (Appendix Figure 1). The introduction of the new clade, DV1-NI-2, and its cocirculation with the longer-standing Nicaragua-endemic clade, DV1-NI-1, represent increased DENV-1 genetic diversity circulating in Nicaragua in 2022.

### DENV-2

**Figure 3:**
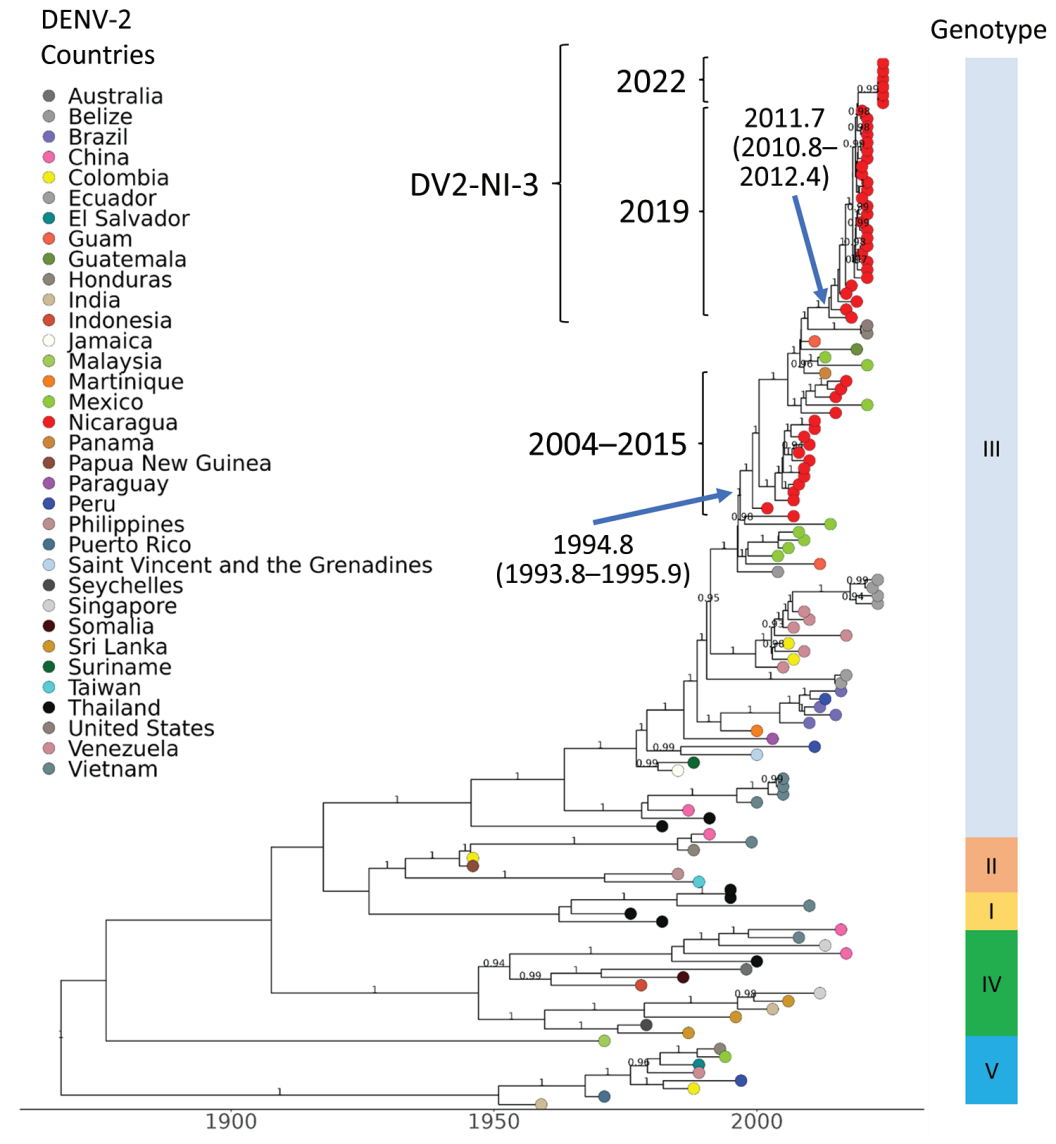
Time-scaled dengue virus (DENV) serotype 2 maximum clade credibility tree, generated from 136 DENV-2 nucleotide sequences, showing emergence over time. Sequences are labeled by country of sample origin, indicated by colored circles, and include Nicaragua sequences (red) and publicly available sequences, as indicated. Nicaragua clades are indicated with square brackets labeled by year(s) of detection, including the DV-NI-3 clade. The time to most recent common ancestor (95% high probability density) is indicated at the nodes leading to Nicaragua clades. Genotypes are identified by vertical bar to the right. Posterior node probabilities are indicated at major nodes.

We paired 6 DENV-2 sequences with 130 previously published genome sequences for phylogenetic analysis and tree building as previously described (MCC tree, Figure 3; Appendix Figure 2, showing the initial ML tree with 436 taxa). DENV-2 viruses circulating in Nicaragua in 2022 descended from DENV-2 present in Nicaragua since 2013 (clade DV2-NI-3B) and associated with epidemics in 2016 (*10*) and 2019, the latter marked by elevated numbers of cases, increased levels of disease severity, and accelerated rates of adaptive evolution (*18*). The estimated tMRCA of DV2-NI-3 was 2011.7 (95% HPD 2010.8–2012.4) (Figure 3). Globally, this DENV-2 lineage is a member of the Southeast Asian–American genotype IIIb, the only genotype circulating in Nicaragua since 1999 and in the region since the 1980s (*5*,*10*,*18*).

### DENV-3

We combined the 38 DENV-3 sequences generated in this study with 93 published sequences to produce an MCC tree (Figure 4; Appendix Figure 3, showing the initial ML tree with 253 taxa). DENV-3 sequences from 2022 all fell within the Indian-subcontinent genotype III, the only genotype to have been detected in Nicaragua, first recorded in 1994 (*34*). Our prior studies had captured DENV-3 sequence evolution until 2014 (*10*,*28*) with an estimated tMRCA of 2003.8 (95% HPD 2003.2–2004.5) (Figure 4), after which DENV-3 remained undetected until 2022. In 2013 and 2014, multiple lineages of DENV-3 were circulating (*10*), but none persisted; the DENV-3 viruses sequenced in 2022 cluster most closely with viruses reported from Puerto Rico (2022), Brazil (2023), Cuba (2023), the United States (Florida, 2023), and India (2018) (*35*) and have an estimated tMRCA of 2019.8 (95% HPD 2019.5–2020.2) (Figure 4). The basal sequences to this clade are from southern and Southeast Asia, suggesting that the clade was introduced recently (after 2018) into the region, including Nicaragua, replacing earlier strains of this serotype (*11*).

### DENV-4

**Figure 5:**
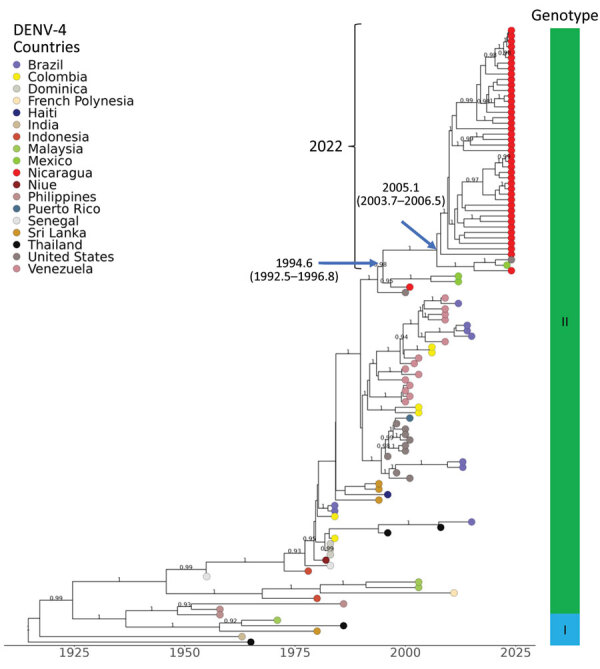
Dengue virus (DENV) serotype 4 maximum clade credibility tree, generated from 115 DENV-4 nucleotide sequences, showing emergence over time. Sequences are labeled by country of sample origin, indicated by colored circles, and include Nicaragua sequences (red) and publicly available sequences, as indicated. Nicaragua clades are indicated with square brackets labeled by year of detection. The time to most recent common ancestor (95% high probability density) is indicated at the nodes leading to Nicaragua clades. Genotypes are identified in the vertical bar to the right. Posterior node probabilities are indicated at major nodes.

We combined 42 DENV-4 sequences with 73 publicly available sequences to generate an MCC tree (Figure 5; Appendix Figure 4, showing the initial ML tree with 168 taxa). DENV-4 has not been detected in dengue patients in Nicaragua since 2006. Our analysis places DENV-4 sequences within genotype II, Indonesia, with an estimated tMRCA of 2005.1 (95% HPD 2003.7–2006.5) (Figure 5). The sequences most closely related to the Nicaragua strains are from Mexico, El Salvador, and Florida (2021–2022). However, the clade includes basal sequences from Mexico (2010) and Nicaragua (1999), suggesting a regionally long-circulating lineage with not-infrequent exchange between countries since 1999 until 2022. From this information, we conclude that there is a regional reservoir and source of DENV-4 into Nicaragua.

## Discussion

In 2022, genomic characterization of DENV in Nicaragua was conducted in real time during an ongoing epidemic. That approach revealed the considerable cocirculation of all 4 DENV serotypes, within the context of a particularly large epidemic in 2022. Phylogenetic analyses of the 2022 viruses enabled us to identify the introduction of new DENV strains (DENV-1, DENV-3, DENV-4) from different regions of the Americas, although a small number of DENV-1 and DENV-2 viruses remained from previous periods of circulation in Nicaragua. We noted the resurgence of serotypes not seen since 2013 (DENV-1 and DENV-3) and the introduction of DENV-4, which had not circulated at epidemic levels since the early 1990s (*10*,*18*).

Because of the emergence of SARS-CoV-2 in 2019 and the subsequent COVID-19 pandemic, many countries closed their borders, restricted mobility, and implemented social distancing (*36*). Nicaragua was one of the few countries to use alternative approaches to shutdowns (i.e., travel restrictions and neighboring border closures). Vector control activities (e.g., residual indoor and spatial fumigation) were maintained. After 2 years of low dengue case incidence in 2020 and 2021, dengue cases in Nicaragua increased substantially during 2022, marked by the cocirculation of all 4 virus serotypes. We suggest that the marked dominant circulation of DENV-2 over the previous 7 years contributed strongly to the sudden rise of DENV-1, DENV-3, and DENV-4 cases in 2022 after new introductions (*11*,*18*,*37*). The absence of herd immunity to all serotypes except DENV-2, coupled with resumption of postpandemic international travel, increased the risk for establishment of new DENV strains in Nicaragua.

Of the 2022 dengue cases in Nicaragua sequenced in our study, 52% were caused by DENV-1, falling into 2 coexisting lineages within genotype V. Although a small number of sequences persisted from earlier 2012–2013 epidemics, 90% formed a new clade (DV1-NI-2) with genetic ties to DENV-1 in Ecuador, Colombia, and Venezuela. Gene flow has been previously reported among those countries (*38*,*39*) and might have been accelerated by post–COVID-19 border openings and regional increases in movement or displacement with South America (*40*), ultimately leading to a turnover of the previous Nicaragua DENV-1 strains. Alternatively, the evolution of clades could represent undetected transmission of DENV-1 in the country over that period, which seems to be the case with DV1-NI-1. However, our results suggest that DV1-NI-2 was introduced after 2013. Pinpointing the exact source and potential influence of neighboring countries such as Costa Rica and Panama, with which Nicaragua shares close migratory ties, is limited because of the lack of public sequences from Central America during the study period.

Previous studies have detailed the clinical, epidemiologic, and immunologic pattern of DENV-2 within the Nicaragua population (*3*,*5*,*10*,*11*,*41*,*42*) (F. Narvaez et al., unpub. data, https://www.medrxiv.org/content/10.1101/2024.02.11.24302393v1). Emergence of the new, more fit, DV2-NI-2B clade (*5*,*43*) in an immunologically susceptible population during 2007–2008 was followed by further adapted DENV-2 lineages associated with outbreaks in 2015–2016 and 2019 (DV2-NI-3) (*10*,*18*). Our analysis confirmed that DENV-2 strains during 2022 descended from these previous strains, within the Southeast Asian–American genotype IIIb. Unlike the other serotypes, evolution of the DV2-NI-3 clade is consistent with a single introduction and subsequent local expansion, with no additional introductions or admixture from other regions, based on publicly available reference sequences. We believe that the recent decrease in DENV-2 cases within the population resulted from development of serotype-specific immunity from many years of almost exclusive apparent circulation of DENV-2. The low immunologic profile of the other serotypes contributed to a change in the disease trend of shifting dominance to serotypes other than DENV-2, as well as increased cases in 2022. Nevertheless, the recent introduction of the cosmopolitan DENV-2 genotype into Peru in 2019 (*44*) and Brazil in 2021 (*45*,*46*) poses a new risk for the country. As herd immunity for DENV-2 wanes with time, the introduction of a new genotype of DENV-2 associated with greater severity could increase both the incidence and severity of future cases. That possibility highlights the value of strong and continued genomic surveillance of DENV to determine in real time the genetic diversity and possible correlation with severity in future epidemics in Nicaragua.

Starting with the large 1994 dengue epidemic that was triggered by the initial introduction of DENV-3 into Nicaragua (*47*), DENV-3 has continued to cause epidemics (*14*,*28*). Dominating during 2008–2011, including a major epidemic in 2009–2010 (*28*), DENV-3 dropped noticeably in 2013–2014 (*10*). The 2013–2014 viruses were associated with earlier DENV-3 lineages circulating in Nicaragua (*10*), whereas our analysis reveals that 2022 DENV-3 strains, while still belonging to genotype III/Indian subcontinent, are part of a new, genetically distinct, monophyletic clade, replacing the previous variants. The clustering of the 2022 DENV-3 clade with viruses from India (2018), Puerto Rico (2022), and Brazil-Cuba-Florida (2023) implicates a recent introduction into the region, similar to DENV-1, possibly resulting from opened borders and increased international travel, and may represent an emerging epidemiologic situation of ongoing exchange between countries in the Caribbean, Central America, and South America.

In Nicaragua, DENV-4 caused epidemics in the early 1990s and has been virtually unreported since, with the exception of a few cases in 2006–2008, despite reports of DENV-4 circulation in South America in 2010 (*48*). In 2022, DENV-4 reemerged in Nicaragua, codominating the epidemic with DENV-1. That phenomenon could be attributed to low herd immunity resulting from limited DENV-4 circulation over the previous 30 years, coupled with increased cross-border and domestic movement leading to local virus transmission of what is considered the mildest of the 4 serotypes (*7*). DENV-4 sequences recovered in 2022 clustered with viruses from Florida, El Salvador, and Mexico collected in the same time frame, although the clade does have roots in Nicaragua (1999) as well as the region (e.g., Mexico 2010), suggesting resurgence of a long-standing lineage endemic to regions in Central America and the Caribbean.

We note limitations of this research in inferring chains of transmission within and across geographic regions, which would require systematic sampling of the populations in question—a resource-intensive undertaking involving the collaboration of epidemiologists, health services, and national virology laboratories. In the absence of comprehensive sampling, the issue of bias was addressed by random sampling for sequencing by the laboratory, stratified by geographic source within the country, without knowledge of the clinical status of the patient. We acknowledge that our phylogenetic inferences are biased by the sequence data available in the public domain, as with any phylogenetic analysis accessing publicly available sequences. Last, we did not assess the contribution of mosquito vectors to the transmission dynamics of dengue viruses. Nonetheless, our data offer valuable insights that will be beneficial for future research locally and regionally.

We report a new landscape of DENV transmission in Nicaragua, marked by the cocirculation of all 4 serotypes along with intensifying epidemic magnitude. Of the 4 DENV serotypes, 3 resurged after almost a decade or more of low to no case reports and coincide with the introduction of new lineages. We posit that the dengue hyperendemicity is the result of 2 windows of opportunity: a vacuum of herd immunity against DENV-1, DENV-3, and DENV-4 resulting from the prolonged dominance of DENV-2; and a surge of regional and international travel on the heels of COVID-19-induced country-level and worldwide mobility restrictions (*49*). The landscape of DENV transmission has been reset to include the active circulation of all 4 serotypes after the 2016 introduction of Zika virus, which is known to engender DENV cross-reactive immune responses with epidemiologic and clinical consequences (*11*,*12*). Our findings underscore the urgent need for public health interventions aimed at controlling the spread of dengue in Nicaragua and beyond, particularly in light of the increasing intensity of dengue epidemics and the potential for further transmission fueled by regional and international travel.

Appendix Additional information for study of introduction of new dengue virus lineages after COVID-19 pandemic, Nicaragua, 2022.
