# Supplementary material for: Introduction of New Dengue Virus Lineages of Multiple Serotypes after COVID-19 Pandemic, Nicaragua, 2022
Source: Emerg Infect Dis. 2024 Jun;30(6):1203–13. doi: 10.3201/eid3006.231553 (PMC11138998; doi:10.3201/eid3006.231553)
Supplement: Appendix — Additional information for study of introduction of new dengue virus lineages after COVID-19 pandemic, Nicaragua, 2022. [file 23-1553-Techapp-s1.pdf]

Article DOI: https://doi.org/10.3201/eid3006.231553

EID cannot ensure accessibility for supplementary materials supplied by authors. Readers who have difficulty accessing supplementary content should contact the authors for assistance.

Introduction of New Dengue Virus Lineages after COVID-19 Pandemic, Nicaragua, 2022

Appendix

Appendix Table. Sources of the samples sequenced from the 2022 dengue season in Nicaragua

	Study sites			
Sequences	**A2CARES (n=4) n (%)***	**PDHS (n=13) n (%)†**	**PDCS (n=57) n (%)‡**	**MINSA (n=61) n (%)§**
DENV-1 genotype V (n=49)	2 (4.1)	8 (16.3)	10 (20.4)	29 (59.2)
DENV-2 Asian/ American genotype IIIb (n=6)	Not applicable	Not applicable	6 (100.0)	Not applicable
DENV-3 Indian sub-continent genotype III (n=38)	Not applicable	1 (2.6)	35 (92.1)	2(5.3)
DENV-4 genotype II (n=42)	2 (4.8)	4 (9.5)	6 (14.3)	30 (71.4)

*A2CARES, American-Asian Arbovirus Research and Epidemiological Surveillance cohort study.
†PDCS, Pediatric Dengue Cohort Study.
‡PDHS, Pediatric Dengue Hospital-based Study.
§MINSA, Nicaragua Ministry of Health surveillance.

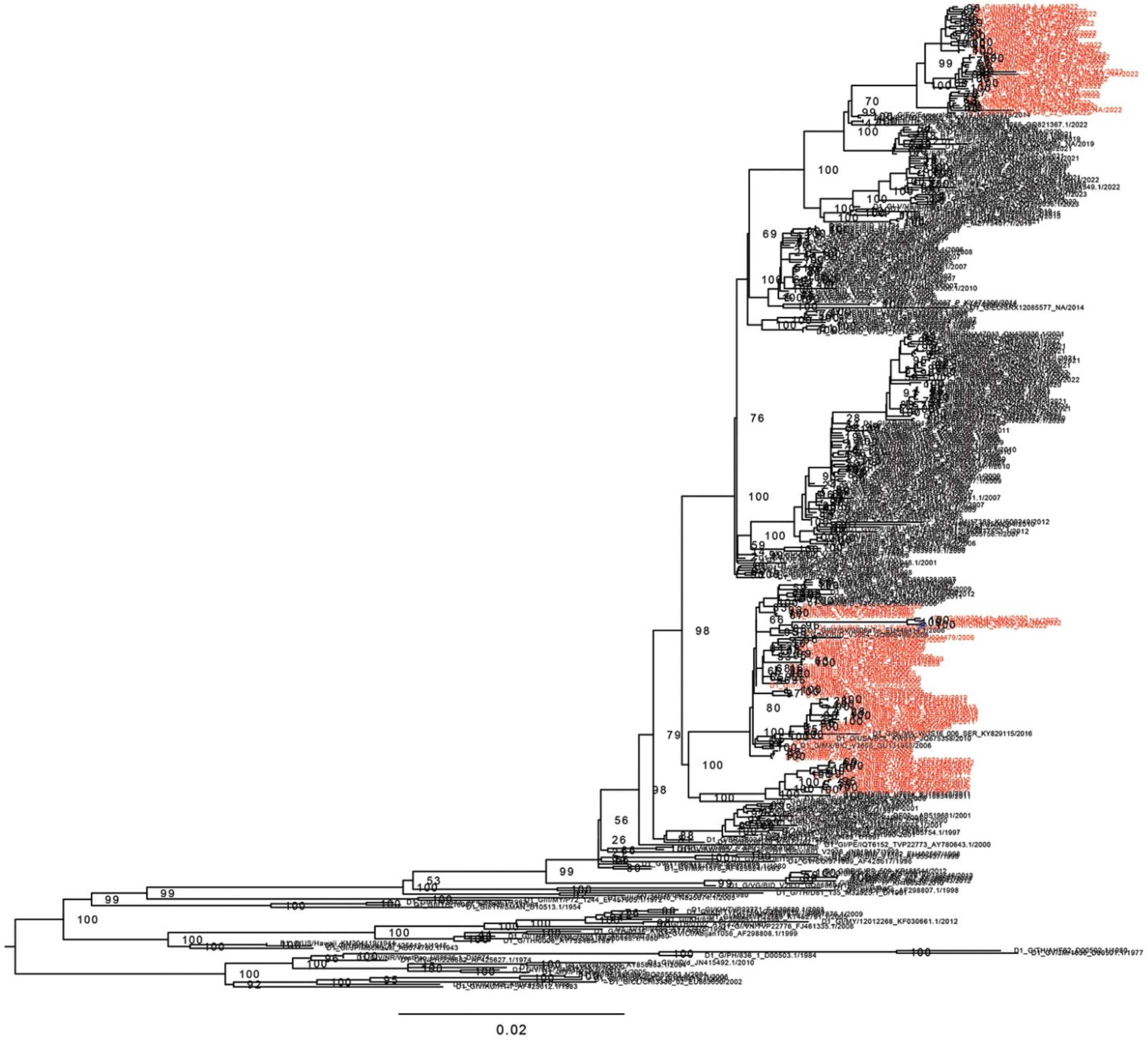

Appendix Figure 1. Maximum Likelihood tree of dengue virus serotype 1 generated in RAxML-HPC BlackBox v.8.2.12 based on an alignment of 408 sequences including those contributed by this study as well as publicly available sequences from NCBI Virus via BLAST and a comprehensive search by region over time. Nicaraguan sequences colored red. Nodes are labeled with ML bootstrap support values (where default settings were used: RAxML halts bootstrapping automatically when convergence is achieved). Scale bar in substitutions/site.

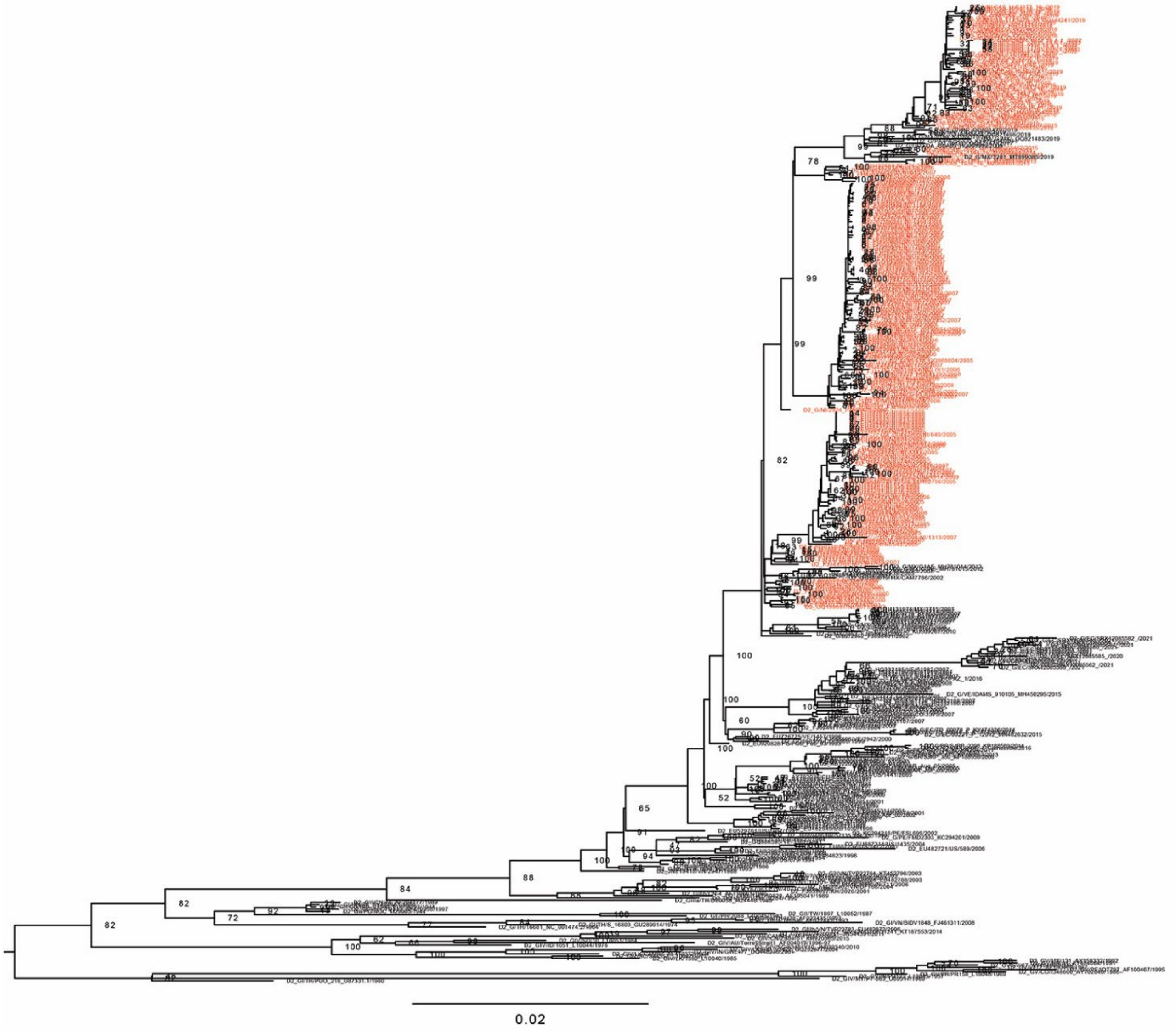

Appendix Figure 2. Maximum Likelihood tree of dengue virus serotype 2 generated in RAxML-HPC BlackBox v.8.2.12 based on an alignment of 436 sequences including those contributed by this study as well as publicly available sequences from NCBI Virus via BLAST and a comprehensive search by region over time. Nicaraguan sequences colored red. Nodes are labeled with ML bootstrap support values (where default settings were used: RAxML halts bootstrapping automatically when convergence is achieved). Scale bar in substitutions/site.

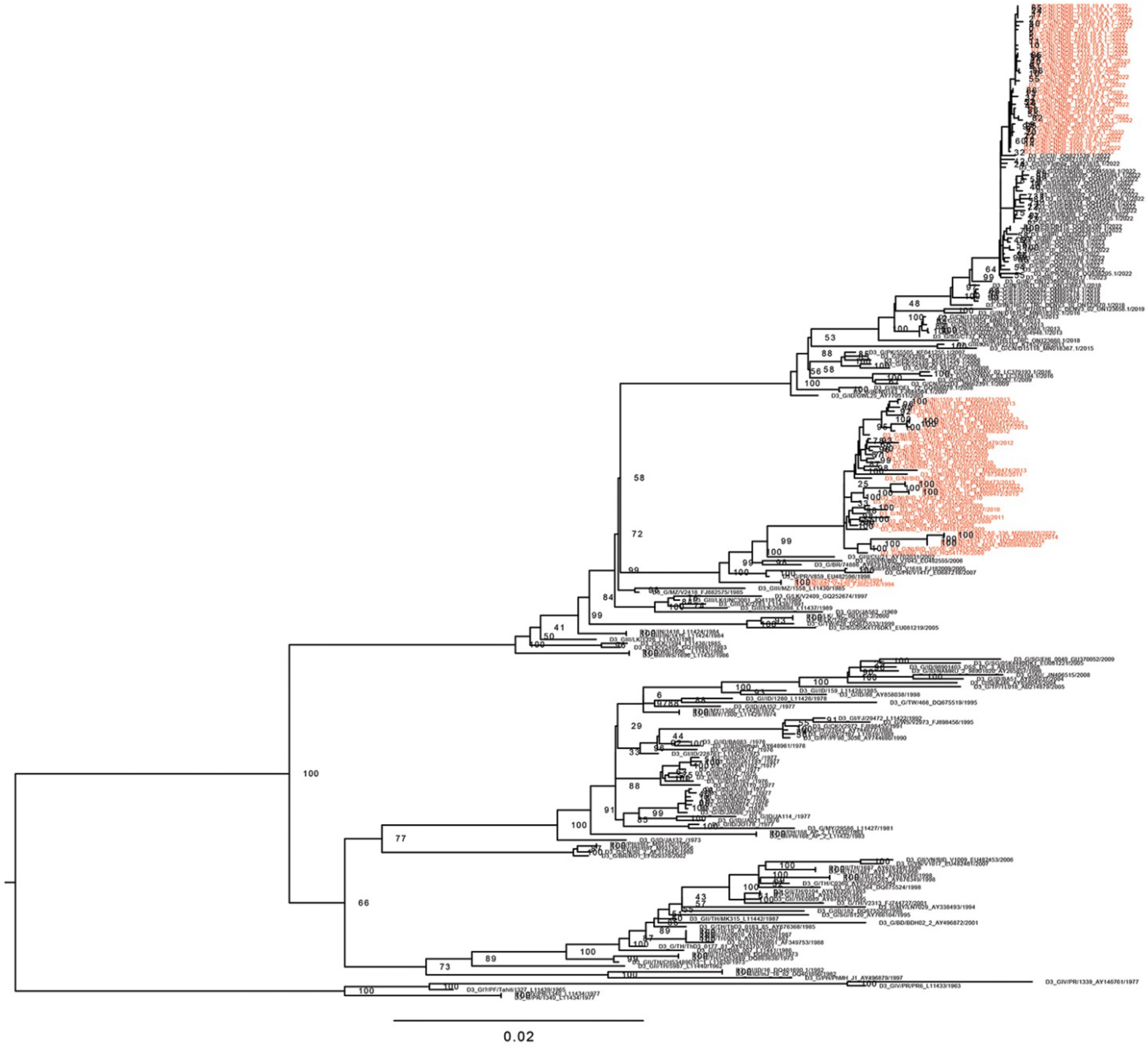

Appendix Figure 3. Maximum Likelihood tree of dengue virus serotype 3 generated in RAxML-HPC BlackBox v.8.2.12 based on an alignment of 253 sequences including those contributed by this study as well as publicly available sequences from NCBI Virus via BLAST and a comprehensive search by region over time. Nicaraguan sequences colored red. Nodes are labeled with ML bootstrap support values (where default settings were used: RAxML halts bootstrapping automatically when convergence is achieved). Scale bar in substitutions/site.

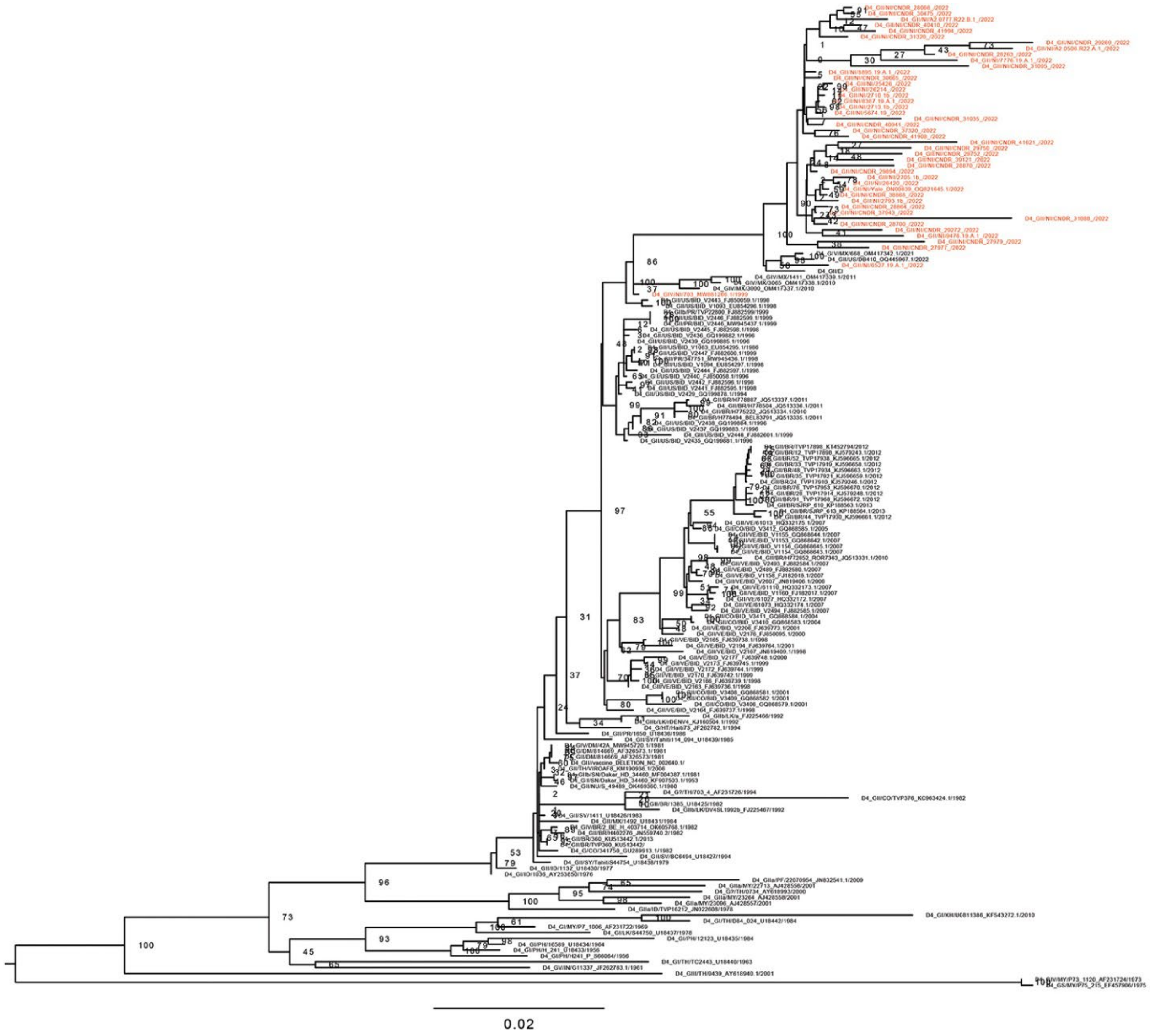

Appendix Figure 4. Maximum Likelihood tree of dengue virus serotype 4 generated in RAxML-HPC BlackBox v.8.2.12 based on an alignment of 168 sequences including those contributed by this study as well as publicly available sequences from NCBI Virus via BLAST and a comprehensive search by region over time. Nicaraguan sequences colored red. Nodes are labeled with ML bootstrap support values (where default settings were used: RAxML halts bootstrapping automatically when convergence is achieved). Scale bar in substitutions/site.
